# Prevención del suicidio en el entorno laboral

**DOI:** 10.1016/j.aprim.2024.103145

**Published:** 2024-11-19

**Authors:** Israel Macías-Toronjo, Juan Jesús García-Iglesias, Juan Gómez-Salgado

**Affiliations:** a Departamento de Rehabilitación, Hospital Fremap Huelva, Huelva, España; b Departamento de Sociología, Trabajo Social y Salud Pública, Universidad de Huelva, Huelva, España; c Universidad Espíritu Santo, Guayaquil, Ecuador

Todos los años fallecen en el mundo 800.000 personas por suicidio, según la Organización Mundial de la Salud (OMS). En España, el número de suicidios, a pesar de haber disminuido levemente en este último año, se sitúa como la segunda causa de muerte externa, con alrededor de 4.000 muertes anuales^1^. En perspectiva, en España mueren 10 personas al día por suicidio, más del doble que las producidas por accidentes de tráfico.

La etiología del fenómeno suicida es considerada multifactorial. El modelo volitivo motivacional integrado (VMI) del comportamiento suicida de O’Connor^2^ describe un diseño biopsicosocial de tres fases donde subyacen factores biológicos, genéticos o cognitivos o características individuales diferenciales que elevan el riesgo de suicidio y que interaccionan con eventos negativos que podrían desencadenar el acto suicida. Los factores laborales estarían incluidos dentro de este tipo de factores psicosociales desencadenantes. Estudios recientes señalan la creciente importancia de las condiciones laborales en este fenómeno, siendo las profesiones de ciencias sociales y sanitarias en mujeres, y profesiones relacionadas con la industria y las fuerzas de seguridad en hombres, las que habrían aumentado su incidencia^3^. En términos generales, ciertas profesiones con baja cualificación tendrían un mayor riesgo de suicidio que las más cualificadas. En este sentido, los factores intralaborales que podrían aumentar este riesgo incluyen el estrés por una demanda de trabajo excesiva, conflictos en el lugar de trabajo, violencia lateral, sensación de sentirse insuficientemente preparado para el rol, presión para la consecución de objetivos, falta de control sobre la propia vida profesional, violencia en el lugar de trabajo, falta de apoyo profesional por parte de colegas o jornadas de trabajo largas^4^.

En el ámbito ocupacional existen programas específicos que inciden sobre la salud mental que han demostrado tener efectos positivos sobre la ansiedad, el estrés y la depresión, destacando especialmente aquellas que utilizan técnicas de cibersalud y técnicas cognitivas conductuales. De forma más específica, numerosos programas en países de nuestro entorno han incidido sobre la prevención del suicidio desde diferentes abordajes, siendo difícil discernir cuál de ellos tendría un impacto mayor sobre el riego de suicidio^5^. En este sentido, intervenciones basadas en la educación y en la identificación de signos de estrés, enfermedad mental y comportamientos suicidas serían abordajes que podría ser extrapolados a los diferentes contextos ocupacionales y que tendrían un efecto más positivo en el objetivo de disminuir el riesgo suicida^5^. En nuestro entorno, pocas son las intervenciones en el ámbito del trabajo que incluyan la prevención del suicidio dentro de los programas de prevención de riesgos laborales. Si bien es cierto que la identificación y la prevención de problemas de índole psicosocial se han convertido en un elemento central de los programas de prevención, y que la literatura científica ha vinculado los problemas derivados del trabajo con el aumento del riesgo suicida, no existen muchas iniciativas específicas para identificar y reducir el riesgo de suicidio en el ámbito ocupacional. Por otra parte, también recientemente, varias sentencias judiciales han relacionado directamente el suicidio de trabajadores con eventos ocurridos en el plano laboral.

Por tanto, se hace necesario mejorar la comunicación sobre el suicidio para cambiar el prisma a través del cual el suicidio ha sido tratado por medios de comunicación, para que sea abordado desde una perspectiva profesional, sin indagar ni transmitir aspectos que poco tienen que ver con el proceso emocional que engloba, y más con aspectos anecdóticos y morbosos del mismo, con el objetivo de cambiar la percepción de tabú del suicidio y de normalizar la comunicación sobre problemas psicoemocionales. Incluir el abordaje de la salud mental y el suicidio dentro de programas de prevención laborales trata de desmitificar y normalizar este fenómeno, mejorando la comunicación y la sensibilización hacia el mismo^6^.

La salud mental en general y el suicidio en particular deberían trascender el ámbito meramente sanitario e iniciar un camino hacia la salud comunitaria y la salud laboral, incorporando a otros agentes sociales donde la empresa debe estar de manera protagonista^7^. Estas deberían incluir la salud mental dentro sus planes estratégicos, y que estos vayan de la mano de la cultura de empresa al tiempo que se identifican con ella. Para ello, son necesarios planes estratégicos que incluyan programas de prevención de riesgos psicosociales que incorporen el apoyo emocional de los trabajadores, que sean capaces de diagnosticar e identificar a tiempo a personas en riesgo psicosocial y con ideación de suicidio, y donde los trabajadores tengan puntos de apoyo a los que acudir cuando afronten momentos de conflicto emocional.

La efectividad de los programas en ámbito laboral sobre la prevención del suicidio tiene todavía mucha capacidad de mejora^7^. Existen estudios que indican que las pocas iniciativas evaluadas en este sentido tienen resultados beneficiosos, pero que estas necesitan estar integradas en protocolos de salud mental en el trabajo, al tiempo que se necesitan más estudios y actividades de este tipo para el desarrollo, la implementación y la evaluación de este tipo de actividades^8^. La integración dentro de los planes de prevención psicosocial se hace fundamental para que estas iniciativas sean específicas en función del ámbito de trabajo, para mejorar la comunicación y la identificación de trabajadores inmersos en un proceso de ideación suicida.

Teniendo en cuenta que los problemas relacionados con la salud mental constituyen ya la segunda causa de baja laboral en España, y que el suicidio es la primera causa de muerte externa en el grupo de edad que va de los 15 a los 29 años, franja que suele coincidir con la incorporación a la vida laboral, es esencial la toma de conciencia por parte de agentes sociales de este problema de salud pública. Estos datos indican el sentido hacia dónde se deben dirigir las iniciativas y los esfuerzos, donde la empresa debe sentirse partícipe y responsable teniendo en cuenta el peso que tiene el ámbito ocupacional en la vida de los trabajadores y su capacidad para poner en marcha campañas de sensibilización y visibilización. Para ello, se pueden incluir protocolos de prevención y cribado específicos del suicidio dentro la vigilancia de la salud en reconocimientos médicos, generando redes de apoyo entre los trabajadores que podrían ser transversales, creando grupos de primeros auxilios emocionales que ya han sido implementados en otros países con buenos resultados, y favoreciendo, en definitiva, entornos de trabajo saludables y protectores donde la salud en general y la salud mental de forma específica sean consideradas un proyecto compartido por trabajadores y empresas.

## Financiación

Este estudio no ha recibido financiación alguna, por parte de organismos públicos ni privados, para su desarrollo.

## Contribuciones de los autores

Conceptualización: IMT y JGS. Curación de datos: IMT y JJGI. Análisis formal: IMT, JGS y JJGI. Adquisición de fondos: JGS. Investigación: IMT, JGS y JJGI. Metodología: IMT, JGS y JJGI. Administración de proyectos: JGS y JJGI. Software: IMT. Recursos: IMT, JGS y JJGI. Supervisión: JJGI. Validación: JGS. Visualización: IMT, JGS y JJGI. Redacción - borrador original: IMT y JGS. Redacción - revisión y edición: JJGI.

## Conflicto de intereses

Los autores de esta investigación no tienen conflicto de interés, y lo planteado en la investigación es fruto del análisis de los resultados obtenidos.
